# Prevalence of HIV and Associated Risks of Sex Work among Youth in the Slums of Kampala

**DOI:** 10.1155/2016/5360180

**Published:** 2016-04-28

**Authors:** Monica H. Swahn, Rachel Culbreth, Laura F. Salazar, Rogers Kasirye, Janet Seeley

**Affiliations:** ^1^School of Public Health, Georgia State University, P.O. Box 3995, Atlanta, GA 30302-3995, USA; ^2^Uganda Youth Development Link, P.O. Box 12659, Kampala, Uganda; ^3^London School of Hygiene and Tropical Medicine, Keppel Street, London WC1E 7HT, UK

## Abstract

*Purpose*. The purpose of this study is to examine the prevalence of and risk factors for engaging in sex work among youth living in Kampala, Uganda.* Methods*. Analyses are based on a cross-sectional study (*N* = 1,134) of youth aged 12-18 years, living in the slums of Kampala, conducted in Spring of 2014. The analytic sample consisted of only sexually active youth (*n* = 590). Youth who reported engaging in sex work were compared to youth who did not report sex work. Multivariable analyses were conducted to examine factors associated with sex work.* Results*. Among the youth who had ever had sexual intercourse (*n* = 590), 13.7% (*n* = 81) reported engaging in sex work. Self-reported HIV prevalence was 13.9% among the total sample (*n* = 81) and 22.5% (*n *= 18) among youth engaged in sex work. Engaging in sex work was associated with being female (AOR 10.4; 95% CI: 3.9, 27.4), being an orphan (AOR 3.8; 95% CI: 1.7, 8.4), ever drinking alcohol (AOR 8.3; 95% CI 3.7, 19.0), and experiencing any rape (AOR 5.3; 95% CI: 2.9, 9.5).* Discussion*. The reported prevalence of sex work is high among youth in the slums of Kampala and is associated with high HIV prevalence, ever drinking alcohol, previously being raped, and being an orphan.

## 1. Background

HIV prevalence among Ugandan youth aged 15–19 is estimated at 2.4% [1], with much higher rates estimated among vulnerable youth living in the slums, for both HIV and sexually transmitted infections (STIs) (37.2%) [2]. High-risk behaviors such as engaging in commercial sex work in addition to other HIV-related risk behaviors such as multiple sexual partners, intermittent condom use, and concurrent substance use all increase the risk of acquiring and transmitting HIV [3].

Commercial sex work is defined by the United Nations as the “exchange of money or goods for sexual services, either regularly or occasionally, involving female, male, and transgender adults, young people and children where the sex worker may or may not consciously define such activity as income-generating” [4]. Sex work is associated with a number of risk factors, including alcohol and substance use [5–9], adverse childhood experiences, including previously being sexually or physically abused and parental substance abuse [10, 11], and social factors, such as orphan status or a lack of educational training [12–14]. Among a sample of female sex workers in Kampala, 49% reported being raped at least once in their lifetime [6]. Youth who are orphans or those who do not attend school may resort to sex work for food, shelter, and income [12–14].

Additionally, sex work has been linked to numerous adverse health consequences. HIV prevalence among those who engage in sex work is nearly 12 times higher than the general population [15]. A study examining 16 countries in Sub-Saharan Africa reported a 37% HIV prevalence among females engaging in sex work [15]. Clients of commercial sex may request unprotected sex and have reported paying more for this sex [16], further increasing the risk of HIV and other STI acquisition [17].

Sex workers have also reported experiencing violence from clients which may lead to an increased risk of HIV transmission [6, 7, 18, 19]. Among a sample of adult female sex workers in Kampala, Uganda, 82% reported being a victim of violence initiated by the client [6]. Boys and men are also vulnerable to violence when engaging in sex work [20]. Additionally, the use of alcohol before or during sex has been linked to risk for violence in the general population as well as among sex workers [6, 8, 21–25]. Sex workers who use substances such as alcohol also report higher instances of engaging in sex without a condom, further increasing risk of HIV transmission and acquisition [7].

Despite the health needs and disparate circumstances of youth who live on the streets and slums of Kampala, Uganda, and their high risk for engaging in sex work [26, 27], we could not find any study that has examined sex work specifically in this population. The lives of youth living in the slums and streets of Kampala are complicated by many additional adversities due to their living conditions that exacerbate the vulnerability of the youth, such as food scarcity, a lack of money, and abuse and neglect [2, 16, 27–32]. Examining high-risk behaviors among these youth is important to inform interventions which target this vulnerable and growing population. The purpose of this analysis is to estimate the HIV prevalence and associated psychosocial correlates of commercial sex work among youth living in the slums so that prevention and intervention strategies can be designed and implemented.

## 2. Methods

### 2.1. Setting

The current study is based on the “Kampala Youth Survey 2014,” a cross-sectional survey conducted in March and April 2014 to quantify and examine high-risk behaviors and exposures, with a focus on alcohol use, sexual behaviors, and HIV, in a sample of urban youth, 12–18 years of age, living in the slums or on the streets of Kampala, Uganda, who were participating in a Uganda Youth Development Link (UYDEL) drop-in center for disadvantaged street and slum youth [33]. Study participants were recruited at six drop-in centers and the neighborhoods surrounding the UYDEL drop-in centers primarily through word of mouth.

### 2.2. Participant Recruitment/Data Collection

Over the 15-day (March 19 to April 2) data collection period, 1,628 youth were approached for participating in the survey. Among these youth, 131 declined yielding a participation rate of 92%. A total of 1,497 surveys were collected, including 43 pilot cases. Three hundred and twenty (320) surveys were lost due technical issues with the offline server, yielding 1,134 completed surveys for the final analytic sample of youth between the ages of 12 and 18 (44% boys, 56% girls).

Each social worker/peer educator received training on the study methodology and survey questions, and they recruited potential participants among attendants at their specific drop-in center. Each of the survey questions was translated into Luganda (local language) if needed. Face-to-face interviews were conducted by social workers and peer educators employed by UYDEL with previous experience working with youth within the targeted drop-in centers and communities. The survey was administered to the participants on Google Nexus 7 tablets. The use of tablets as an mHealth technology allowed for easier administration of the survey and streamlined data collection. Participants were informed about the study and read (or were read) the consent forms to indicate their willingness to take the survey. All participants provided verbal consent to participate in the study. Youth who “cater for their own livelihood” are considered emancipated in Uganda and are able to provide their own consent for the survey without parental consent. Participation was limited to youth aged 12–18 years present in-person on of the day of the field visit. There were no other exclusion criteria. Recruited youth received a small snack as incentive for participating in the survey. IRB approvals were obtained from Georgia State University and the Uganda National Council for Science and Technology to conduct this study in Kampala.

The Kampala Youth Survey 2014 was based on previously validated quantitative measures to assess alcohol use, violence perpetration and victimization, prevalence of alcohol marketing, sexual behaviors, and mental health among adolescents. Participants were asked for information on demographics, alcohol usage, and perception and beliefs related to alcohol and sex as well as their knowledge of HIV/AIDS and other STIs. Questions included in the survey were collected from previously validated instruments used in the United States and globally to measure alcohol and sexual related behavior as well as mental health, including Global School-Based Student Health Survey (GSHS) [34], Kampala Youth Survey 2011 [2, 16, 27–29], MAMPA 2012 Questionnaire, AUDIT Questionnaire [35], CAGE Questionnaire [36], iMPPACS [37], AIDS Indicator Survey [1], and the Demographic Health Survey [38]. The survey was available to participants in both English and Luganda (the main local language). All survey translation was conducted by a certified Luganda translator and backtranslated for accuracy.

### 2.3. Data Analysis

#### 2.3.1. Dependent Variable

*Commercial Sex Work*. A youth was classified as engaging in commercial sex work if he or she answered “yes” to “are you currently engaged in commercial sex work?”

#### 2.3.2. Independent Variables

*School attendance* was classified into two categories: ever attending school and never attending school.* Parental drunkenness* was assessed by the question “did your parents/caretakers drink a lot of alcohol when you were growing up?” Assessing* family living status* was assessed using “are one or both of your parents alive?” Individuals could answer “both parents are alive,” “both parents are dead,” or “one parent living.”

*Ever drinking alcohol* was measured using “how old were you when you had your first full drink of alcohol?” Individuals were classified as ever having an alcoholic drink if they responded to an age category, and individuals were classified as never having alcoholic drink if they responded with “never.”

*Parental child abuse* was assessed using “did a parent beat you so hard you had bruises or marks?”* Rape prevalence* was measured using “has someone ever raped you or forced you to have sex with him or her?”

Demographic variables included* age, sex, education*, and* religion*.* Age* was categorized into three categories: 12–14 years, 15-16 years, and 17-18 years.* Sex* was dichotomized as male/female.* Education* was classified as “never been to school” and “ever attended school.”* Religion* was categorized as “Christian- Catholic,” “Christian- other,” “Muslim,” and “other.”

#### 2.3.3. HIV Prevalence

Youth were classified as HIV-positive if he or she answered “yes” to “have you ever been told by a doctor/nurse or HIV counselor that you have HIV?” Having a sexually transmitted disease was classified as answering “yes” to “have you ever been told by a doctor/nurse or HIV counselor that you have a sexually transmitted infection?”

#### 2.3.4. Characteristics among Sex Workers

Characteristics among sex workers included age at sex work initiation, payment for sex work, past 3-month client condom use, and reasons for no condom use with a client. Age at sex work initiation was categorized into less than or equal to 12 years, 13-14 years, 15-16 years, and 17-18 years. Payment for sex was classified as money, food, shelter, school fees, transportation, entrance to disco/cinema halls, alcohol, and other reasons. Past 3-month client condom use was categorized into never, sometimes, most of the time, and always. Reasons for no condom use with client were classified into 5 options: when a client pays more, with regular clients, when clients look healthy, other, and never (always use condoms with client). Youth could respond with more than one answer for payment for sex work and reasons for no condom use with a client.

#### 2.3.5. Statistical Analysis

Only youth who reported ever having sex were included in the analysis. Youth who engaged in sex work were compared to youth who did not report engaging in sex work. This exclusion criterion enabled the comparability of the two groups.

Descriptive statistics were computed, including age, sex, education, and religion. Descriptive statistics for characteristics among sex workers were also computed, including age at sex work initiation, payment for sex work, past 3-month client condom use, and reasons for no condom use with client. HIV prevalence and other STI prevalence were computed. Chi-square tests were performed to assess differences in covariates. Bivariate and multivariable analyses using logistic regression were conducted to examine risk factors associated with sex work. Risk factors were included based on empirical evidence. Hypothesized consequences or outcomes associated with sex work, such as HIV/STI variables, were not included as independent variables in the model, and only chi-square statistics were computed to assess differences for these variables. Unadjusted and adjusted odds ratios were obtained with corresponding 95% confidence intervals. The final multivariable model was selected based on statistically significant results from the bivariate analysis. All analyses were conducted using SAS 9.2 (Cary, NC).

## 3. Results

Among youth who had ever had sexual intercourse, the prevalence of sex work was 13.7% (Table 1). The majority of youth engaged in sex work started engaging in sex work at ages 15-16 (47.5%), and the majority accepted money for sex work (97.5%). A large percentage of youth also were paid in alcohol for sex work (40.7%). In terms of condom use, 25.0% of youth reported that they or their clients* always* used a condom, 44.0% reported* sometimes* using condoms, 21.4% reported* most of the time* using condoms, and 9.5% reported* never* using condoms. Reasons for not using condoms included “when a client or customer pays more” (55.5%), “when dealing with a regular customer” (40.7%), or “when a customer looks healthy” (11.1%).

The prevalence of demographic characteristics, risk factors, and HIV/STIs among youth engaged in commercial sex work, as well as the total sample, is presented in Table 2. Youth who reported engaging in sex work were mostly female (93.8%), were 18 years of age at the time of interview (51.8%), and reported being Christian (another denomination than Roman Catholic) (45.7%). A greater proportion of youth engaging in sex work had never been to school compared to youth who did not engage in sex work (16.3% versus 4.6%, resp.). Most youth engaged in sex work reported parental drunkenness (63.0%) compared to youth not engaged in sex work (51.0%). Additionally, ever consuming alcohol was extremely high among youth engaged in sex work (90.0%) compared to youth not engaged in sex work (53.5%) and the total sample (58.4%). This difference was statistically significant (*P* < 0.01). A greater percentage of youth engaged in sex work reported having no parents alive (44.4%) compared to youth not engaged in sex work (21.2%), which was also statistically significant (*P* < 0.01). Youth engaged in sex work had a high prevalence of any rape (67.9%), which was triple the prevalence of rape among youth not engaged in sex work (19.2%) (*P* < 0.01). Parental abuse of youth was also high among youth engaged in sex work (43.2%), but only slightly higher than the total sample (39.4%) (*P* = 0.45). Additionally, HIV prevalence among youth engaged in sex work was 22.5%, much higher than youth not engaged in sex work (12.5%) as well as the total sample (13.9%) (*P* = 0.02). Other STIs were also significantly higher among youth engaged in sex work compared to youth not engaged in sex work (77.8% versus 48.7%, resp.) (*P* < 0.01).

Results from the bivariate and multivariable logistic regression analysis are also presented in Table 2. In the final model, engaging in sex work was associated with being female (AOR 10.4; 95% CI: 3.9, 27.4), being an orphan (AOR 3.8; 95% CI: 1.7, 8.4), ever drinking alcohol (AOR 8.3; 95% CI 3.7, 19.0), and experiencing any rape (AOR 5.3; 95% CI: 2.9, 9.5).

## 4. Discussion

This study presented a very high prevalence of youth who engage in sex work. Additionally, nearly one-fourth of these youth self-reported being HIV-positive, and the majority of youth reported having other STIs. Other risk factors associated with sex work were also very high, including ever being raped, ever consuming alcohol, experiencing parental abuse and parental drunkenness, and never attending school. Youth living in the slums of Kampala are an especially vulnerable population as they may lack supportive social networks and family support and are exposed to a range of risks that exacerbate their disparate living conditions [2, 29]. In addition, these youths have limited access to resources to ameliorate these risks.

Almost all youth who reported engaging in sex work also reported ever using alcohol. Nearly half of those engaging in sex work also stated that they were paid in alcohol. Uganda has very high levels of alcohol consumption per capita [39] but few prevention and intervention strategies to address alcohol use, particularly among youth. Based on the current findings, it is clear that alcohol and sex work are strongly linked and need to be addressed more specifically in these key populations. Moreover, interventions which address alcohol use among youth could also empower youth and facilitate the uptake of vocational skills and education which in turn may provide other means of work, financial support, and empowerment. Specifically, stricter enforcement of the minimum legal drinking age for consumption of alcohol (which is 18 years in Uganda) as well as entrance into bars and disco halls is needed to prevent initiation of alcohol use and continued consumption. These venues may also be sources of clientele for sex work.

Being an orphan was also statistically significantly associated with engagement in sex work. Youth who are orphans face unique additional challenges, which may include a lack of nurturing and support from caretakers, food and shelter insufficiencies, and a lack of supervision [2]. Being an orphan could lead to youth living on the streets or slums, which expose youth to other high-risk behaviors, such as engaging in sex work. Nearly all of the youth report being paid by money for sex, which indicates the youth relying on sex work as a source of income for survival. A higher proportion of youth engaging in sex work also report never attending school, compared to youth who report not engaging in sex work. Vocational programs and interventions aimed at providing social support and youth employment may provide alternatives for the youth most vulnerable and at risk for engaging in sex work.

Youth who engaged in sex work reported a very high prevalence of experiencing any rape, which was triple the prevalence of rape among youth not engaged in sex work. The youth who are engaged in sex work may need intensive, trauma-informed counseling as part of rehabilitation programs. Experiencing any rape was also associated with a 5 times higher likelihood of engaging in sex work, which helps inform sexual violence interventions.

Although most of the youth engaged in sex work in our study were females, there were also a few males (*n* = 5) who reported engaging in sex work. The majority of sex work literature in Sub-Saharan Africa is focused on female sex workers [40], and few studies have primarily examined male youth who engage in sex work [41, 42]. The inclusion of males engaged in sex work is an important strength of our study. Most male sex workers' clients are also male [40], and this presents a unique challenge in Uganda due to potentially volatile attitudes towards same-sex relations and antihomosexuality laws. Our sample of male adolescents was too small to perform a subgroup analysis; however, future studies should aim to include a larger sample of youth engaged in sex work with efforts to include boys and young men.

It should be noted that the total sample consisted of youth aged 12–18, which raises the concern of child sex trafficking. Although the circumstances regarding the initiation of sex work among these youths are not ascertained from this study, the majority of youth reported initiating sex work at ages 15-16 years. Moreover, sex work initiation through a trafficker is likely one form of sex work initiation among the youth. Uganda created a Prevention of Trafficking in Persons Act (PTIP) in 2009, which punishes individuals convicted of trafficking children for sex or labor; however, only 293 investigations into child sex trafficking occurred in 2014 with only 23 prosecutions and 4 people convicted [43]. A National Taskforce for the prevention and treatment of trafficked victims exists in Uganda, although their efforts could be drastically strengthened [43]. The National Taskforce primarily relies on nongovernmental organizations (NGOs) to provide and coordinate services. Youth living on the streets that are rescued from trafficking situations are often placed in a juvenile detention center, which provides food, shelter, counseling, and medical treatment; however, these centers lack adequate funding and resources [43]. Additionally, the placement of trafficked youth in juvenile detention centers does not contribute to the decriminalization movement of sex workers, and alternative places of treatment are needed for these youth. In 2014, the Ugandan government also aided in the creation of a child help hotline which provides an opportunity to report cases of child sex trafficking to government officials [44]. Other interventions also provide resources and treatment for youth involved in sex work and child sex trafficking. UYDEL, where this study was conducted, has a program specifically for victims of sex trafficking and youth engaged in sex work [33]. This program provides counseling, vocational training, and linkages to medical treatment and other resources to help aid in the rehabilitation of sex trafficking victims. Community and police officials are also trained at UYDEL programs to increase awareness of sex work and sex trafficking [33]. The Skilling Uganda Program and Youth Venture Fund are programs aimed at improving employment among youth to provide an alternative income rather than sex work [44].

While certain programs exist in Uganda to prevent sex work and sex trafficking, there are considerable improvements that need to be made. Improved referrals of sex trafficked children to appropriate child protective services, more accurate identification of child sex trafficking victims, and more coverage of antitrafficking campaigns are needed [43]. Additionally, the majority of services for child sex trafficking victims were noted to be poor, and the majority were targeted only to women and girls [43]. Street children are particularly vulnerable to sex trafficking, and sustainable and accessible interventions are desperately needed to keep children off the streets and away from traffickers [43].

### 4.1. Limitations

Due to the vulnerabilities of these youths, the data collected contain important information that can guide prevention and intervention efforts even though the results may not be generalizable to other populations. Because this study used a cross-sectional study design, temporal and causal relationships between variables cannot be determined. Future studies should consider assessing a temporal relationship between sex work and other risk factors and adverse health consequences to provide more detailed guidance for prevention and intervention strategies. Because HIV/STIs are self-reported in this survey, the HIV/STI prevalence is likely an underestimate of the true prevalence in this population. The timing of HIV acquisition or the source of HIV also cannot be determined form this survey, as some youths may have been born with HIV. Other factors, such as the low literacy rates, resulted in the interviewers reading aloud the survey questionnaire to participants which could also lead to biases, in particular underreporting of high-risk behaviors.

### 4.2. Implications

The prevalence of youth engaging in sex work in the slums in Kampala is high, and these young people are in dire need of interventions which address these complex health risks.

From a public health perspective, the high HIV prevalence among the youth engaging in sex work is a significant cause for concern. Targeted interventions for youth engaging in sex work could substantially impact the HIV/STI prevalence of the youth as well as their clientele. The large percentage of youth who report being paid in alcohol also warrants targeted interventions. Similarly, stricter enforcement of child sex trafficking laws and strengthening current programs are needed to prevent and treat youth involved in sex trafficking and sex work. Interventions should be trauma-informed, due to high rates of violence and other adverse experiences these youths have faced. But perhaps most important will be strategies that encourage and facilitate basic education for these girls and young women so that they have options, are empowered, and can access health information to protect themselves. Finally, it is very clear based on the findings of our study that this population has substantial unmet health needs that need to be addressed more proactively to support them and ameliorate adverse experiences and living context. Most urgent is the need to address the HIV transmission risk which appears to be fueled largely by alcohol use. As such, more alcohol prevention strategies are needed to delay alcohol use initiation and to reduce alcohol use and its role in HIV transmission and other alcohol-related harm.

## Figures and Tables

**Table 1:** Characteristics among youth engaged in sex work (*n* = 81).

	Prevalence *n* (%)
	81 (13.7)

Age at sex work initiation	
≤12	5 (6.1)
13-14	18 (22.0)
15-16	39 (47.5)
17-18	20 (24.4)

Payment for sex work	
Money	79 (97.5)
Food	27 (33.3)
Shelter	17 (21.0)
School fees	1 (1.2)
Transportation	13 (16.0)
Entrance to disco/cinema halls	8 (9.9)
Alcohol	33 (40.7)
Other	11 (13.6)

Past 3-month client condom use	
Never	8 (9.5)
Sometimes	37 (44.0)
Most of the time	18 (21.4)
Always	21 (25.0)

Reasons for no condom use with client	
When client pays more	45 (55.5)
With regular clients	33 (40.7)
When clients look healthy	9 (11.1)
Other	8 (9.9)
Never (always use condoms with clients)	18 (22.2)

Note: youth could select several options for payment for sex and reasons for no condom use; therefore, percentages do not sum to 100%.

**Table 2:** Bivariate and multivariable associations between sex work and covariates among youth living in the slums of Kampala (*n* = 590).

Variable	Sex workers Yes *n* (%)	Sex workers No *n* (%)	Total sample *n* (%)	Unadjusted OR (95% CI)	Adjusted OR (95% CI)	*P*
	81 (13.7)	509 (68.3)	590 (100)			
Age						
12–14 years	4 (4.9)	27 (5.3)	31 (5.3)	Ref	—	0.67
15-16 years	15 (18.5)	116 (22.8)	131 (22.2)	0.9 (0.3–2.8)		
17-18 years	62 (76.5)	366 (71.9)	428 (72.5)	1.1 (0.4–3.4)		

Sex, *n* (%)						
Females	76 (93.8)	271 (53.2)	347 (58.8)	Ref	Ref	*∗∗*
Males	5 (6.2)	238 (46.8)	243 (41.2)	**13.4 (5.3–33.5)**	**10.4 (3.9–27.4)**	

School attendance						
Yes	67 (83.7)	480 (94.3)	547 (93.8)	Ref	Ref	*∗∗*
No	13 (16.3)	23 (5.7)	36 (6.2)	**4.1 (2.0–8.4)**	2.2 (0.8–5.7)	

Religion						
Christian Catholic	28 (34.6)	196 (38.5)	224 (38.0)	Ref	—	0.09
Christian (other)	37 (45.7)	169 (33.2)	206 (34.9)	1.5 (0.9–2.6)		
Muslim	11 (13.6)	118 (23.2)	129 (21.9)	0.7 (0.3–1.4)		
Other	5 (6.2)	26 (5.1)	31 (5.3)	1.3 (0.5–3.8)		

Parental drunkenness						
Yes	51 (63.0)	259 (51.0)	310 (52.6)	1.6 (1.0–2.7)	—	0.05
No	30 (37.0)	249 (49.0)	279 (47.4)	Ref		

No parents alive	36 (44.4)	108 (21.2)	144 (24.4)	**5.4 (2.7–10.9)**	**3.8 (1.7–8.5)**	*∗∗*
1 parent alive	33 (40.7)	205 (40.3)	238 (40.3)	**2.6 (1.3–5.2)**	1.7 (0.8–3.6)	
2 parents alive	12 (14.8)	196 (38.5)	208 (35.3)	Ref	Ref	

Ever alcohol use						
Yes	72 (90.0)	271 (53.5)	343 (58.4)	**7.8 (3.7–16.6)**	**8.3 (3.7–19.0)**	*∗∗*
No	8 (10.0)	236 (46.5)	244 (41.6)	Ref	Ref	

Any rape						
Yes	55 (67.9)	98 (19.2)	153 (25.9)	**8.9 (5.3–14.9)**	**5.3 (2.9–9.5)**	*∗∗*
No	26 (32.1)	411 (80.8)	437 (74.0)	Ref	Ref	

Parental abuse of youth						
Yes	35 (43.2)	197 (38.8)	232 (39.4)	**1.8 (1.1–2.9)**	0.8 (0.4–1.4)	0.45
No	46 (56.8)	311 (61.2)	357 (60.6)	Ref	Ref	

HIV^a^						
Yes	18 (22.5)	63 (12.5)	81 (13.9)	—	—	*∗*
No	62 (77.5)	440 (87.5)	502 (86.1)			

Other STI^a^						
Yes	63 (77.8)	248 (48.7)	311 (52.7)	—	—	*∗∗*
No	18 (22.2)	261 (51.3)	279 (47.3)			

Note: *P* value is obtained from chi-square analyses.

^a^HIV and other STIs not included in the logistic regression analyses due to HIV/STI being hypothesized outcomes of commercial sex work instead of risk factors.

^*∗*^ *P* < 0.05.

^*∗∗*^ *P* < 0.01.
